# Cartilage tissue engineering using decellularized biomatrix hydrogel containing TGF-β-loaded alginate microspheres in mechanically loaded bioreactor

**DOI:** 10.1038/s41598-024-62474-5

**Published:** 2024-05-25

**Authors:** Sima Bordbar, Zhen Li, Nasrin Lotfibakhshaiesh, Jafar Ai, Amin Tavassoli, Nima Beheshtizadeh, Letizia Vainieri, Mehdi Khanmohammadi, Forough Azam Sayahpour, Mohamadreza Baghaban Eslaminejad, Mahmoud Azami, Sibylle Grad, Mauro Alini

**Affiliations:** 1 Tissue Engineering Department, School of Advanced Technologies in Medicine, Tehran University of Medical Sciences, Tehran, Iran; 2 Department of Anatomy, School of Medicine, Tehran Medical Sciences Branch, Islamic Azad University, Tehran, Iran; 3 Department of Stem Cells and Developmental Biology, Cell Science Research Center, Royan Institute for Stem Cell Biology and Technology, ACECR, Tehran, Iran; 4 AO Research Institute Davos, Davos, Switzerland; 5 Division of Biotechnology, Faculty of Veterinary Medicine, Ferdowsi University of Mashhad, Mashhad, Iran; 6 Department of Tissue Engineering, Faculty of Advanced Medical Sciences, Tabriz University of Medical Sciences, Tabriz, Iran; 7 Regenerative Medicine Group (REMED), Universal Scientific Education and Research Network (USERN), Tehran, Iran; 8 Biomaterials Group, Materials Design Division, Faculty of Materials Science and Engineering, Warsaw University of Technology, Wołoska 141, 02-507 Warsaw, Poland; 9 Dentistry School, Marquette University, Milwaukee, WI 53233 USA

**Keywords:** Cartilage tissue engineering, Decellularized biomatrix hydrogel, TGF-β1, Alginate microspheres, Mechanical stimuli bioreactor, Regenerative medicine, Biomedical engineering, Translational research

## Abstract

Physiochemical tissue inducers and mechanical stimulation are both efficient variables in cartilage tissue fabrication and regeneration. In the presence of biomolecules, decellularized extracellular matrix (ECM) may trigger and enhance stem cell proliferation and differentiation. Here, we investigated the controlled release of transforming growth factor beta (TGF-β1) as an active mediator of mesenchymal stromal cells (MSCs) in a biocompatible scaffold and mechanical stimulation for cartilage tissue engineering. ECM-derived hydrogel with TGF-β1-loaded alginate-based microspheres (MSs) was created to promote human MSC chondrogenic development. Ex vivo explants and a complicated multiaxial loading bioreactor replicated the physiological conditions. Hydrogels with/without MSs and TGF-β1 were highly cytocompatible. MSCs in ECM-derived hydrogel containing TGF-β1/MSs showed comparable chondrogenic gene expression levels as those hydrogels with TGF-β1 added in culture media or those without TGF-β1. However, constructs with TGF-β1 directly added within the hydrogel had inferior properties under unloaded conditions. The ECM-derived hydrogel group including TGF-β1/MSs under loading circumstances formed better cartilage matrix in an ex vivo osteochondral defect than control settings. This study demonstrates that controlled local delivery of TGF-β1 using MSs and mechanical loading is essential for neocartilage formation by MSCs and that further optimization is needed to prevent MSC differentiation towards hypertrophy.

## Introduction

Articular cartilage defects can be caused by traumatic injury, rheumatoid arthritis, or osteoarthritis, also called degenerative joint disease^1,2^. This tissue has a limited capacity for self-repair after damage, with consequences such as the loss of chondrocyte viability and function and, finally, permanent tissue degeneration^3^. Existing clinical treatments for cartilage defects, including autologous chondrocyte implantation (ACI), micro-fracture, mosaicplasty, and allograft implants, are not successful in fully restoring functional articular cartilage^3,4^. Hence, fibrous tissue, hypertrophic cartilage, or nonfunctional mechanical tissue are often the outcomes^5,6^.

Recently, decellularized ECM materials have become popular because the matrices retain the native structure of cartilage matrix, which provides cells with both mechanical and biochemical signals to promote stem cell fate and ultimately tissue regeneration^7,8^. The ECM materials can inherently induce stem cell differentiation and tissue regeneration, which may be an attractive alternative from both cost and regulatory standpoints^9^. On the other hand, MSCs do not have the potential for chondrogenic differentiation in the absence of chondrogenic stimuli, such as TGF-β or mechanical stimulation^10,11^. Members of the TGF-*β* superfamily have essential roles in cartilage differentiation through autocrine and paracrine signaling^12,13^. In this context, TGF-β1 is essential for cartilage-like tissue formation^14,15^ and several studies have confirmed its essential roles in cartilage tissue differentiation, regeneration, and healing^16,17^.

However, in addition to the chondrogenic stimuli, delivery methods have impressive effects on the final results^18,19^. On the other hand, the alginate-based systems are ideal candidates for bioactive molecule delivery because of their biocompatibility, flexibility in size and shape, and encapsulation efficiency^20^. Furthermore, alginate MSs allow for controlled diffusion rates of macromolecules^20^.

**Figure 1:**
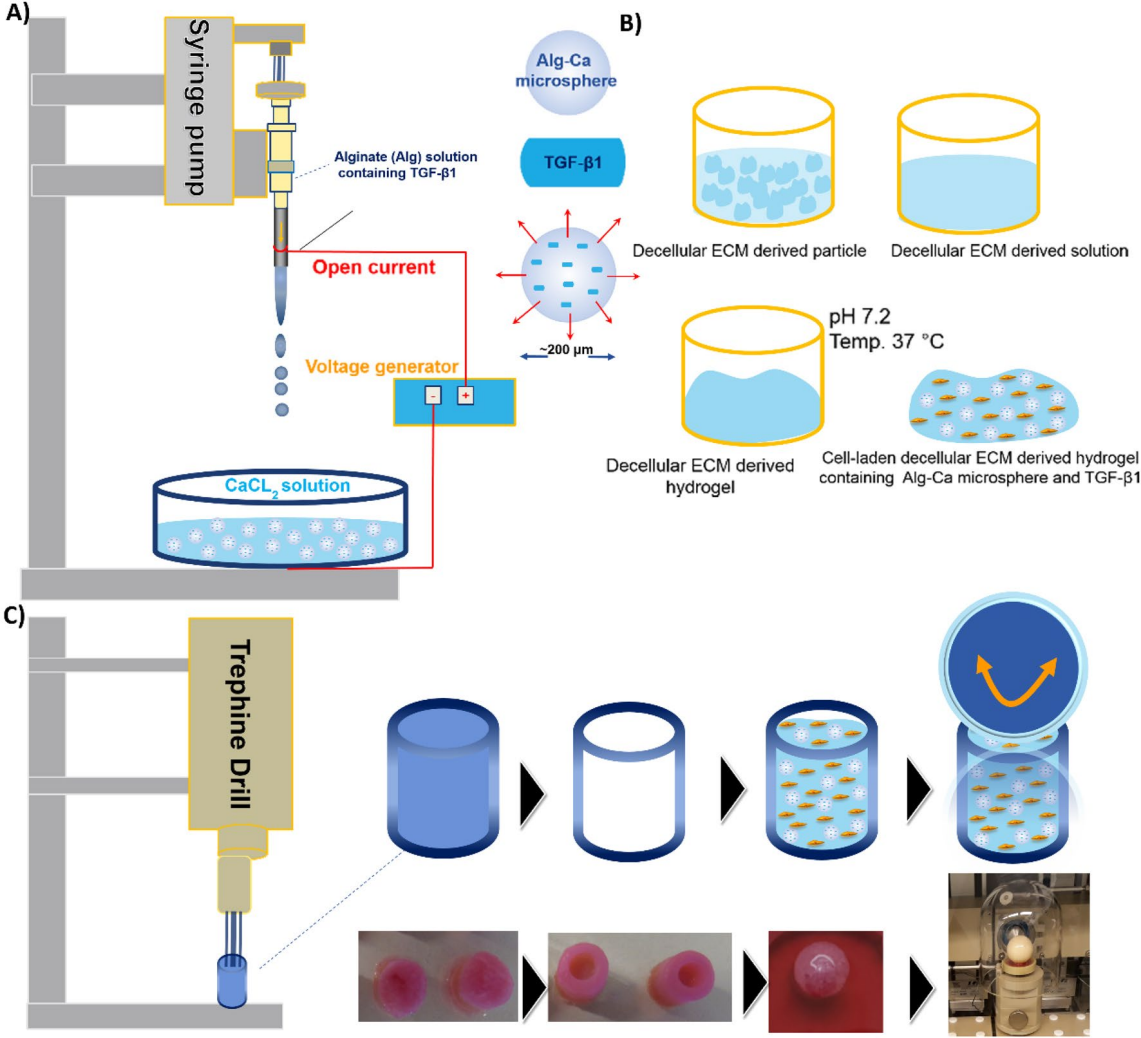
Schematic illustrations of (**A**) production process of Alg–Ca microspheres (MSs) enclosing TGF-β1, and conjugated hydrogel formation through ionic crosslinking between Alg and Ca ions, (**B**) different ECM-derived hydrogels at physiological temperature and pH, and (**C**) production of ex vivo osteochondral plug filled with ECM-derived hydrogels loaded with TGF-β1/Alg–Ca/MSs and MSCs, followed by mechanical stimulation in a complex multiaxial bioreactor.

The objective of this study was to develop an alginate-based delivery vehicle for TGF-β1 and test its efficacy in inducing human MSC chondrogenesis and neocartilage formation, together with decellularized ECM-derived hydrogels in both in vitro and ex vivo* micro*environments^21,22^. The decellularized ECM-derived hydrogel was combined with alginate MSs loaded with TGF-β1 (Fig. 1A) to promote its bioactivity retention in the fabricated hydrogel and stimulate the chondrogenesis of encapsulated MSCs (Fig. 1B). Furthermore, an osteochondral ex vivo explant defect model was used to assess the early cellular responses to complex multiaxial loading in a confined microenvironment via a bioreactor system (Fig. 1C).

## Materials and methods

### Cartilage tissue decellularization

Articular cartilage was harvested from fresh bovine knees obtained from a local slaughterhouse. The femur cartilage was cut into thin, coin-like pieces with a diameter of 5 mm and a thickness of 2 mm. For decellularization, a combination of simultaneous physical and chemical treatments was utilized. For physical decellularization, samples were maintained at − 80 °C for three days, and then the tissue specimen was snap frozen and thawed in liquid nitrogen four times at 2-min intervals. The tissues were pulverized with mechanical force using a Molinex blender. After that, tissues were treated with 5% SDS for 8 h (Merck, Darmstadt, Germany) at 37 °C to achieve proper DNA extraction. Specimens were characterized histologically and by measuring the DNA content. Finally, samples were lyophilized.

### Hydrogel synthesis

In order to synthesize the hydrogel, 1 g of the lyophilized cartilage was dissolved in 100 mL of 0.1 M acetic acid containing 5 mg/mL pepsin for 4 days at 4 °C. After that, the hydrogel was gelled by raising the pH to 7 at 37 °C within 5–7 min^21,22^. Scanning electron microscopy (SEM; AIS2100, Seron Technology, Korea) was used to observe the morphology of decellularized ECM and the hydrogel produced. For this, the specimens were dehydrated through a graded ethanol series (50–95% w/v), sputter-coated with gold (60 mA current, 25 kV, and a duration of 40 s), and examined using SEM.

### Rheometric analyses

Viscoelastic properties were determined within the linear viscoelastic range of the gel. For this, the neutralized ECM-derived hydrogel was placed on a cylindrical module with dimensions of approximately 12 mm in diameter and 6 mm in thickness. Then, it was transferred to the parallel geometric flat probes, and the linear viscoelastic region was measured by performing a stress sweep at a frequency of 1 Hz. The elastic (storage modulus, Gʹ) and viscous (loss modulus, Gʹʹ) components of the sample were measured through a frequency sweep test at 0.01–100% strain with a constant strain detection of 5%.

### Producing alginate-based MS containing TGF-β1

Spherical MSs of 200 µm diameter were generated using an electrostatic droplet generator. For this, 10 and 100 ng TGF-β1 was mixed in 1 mL sodium-alginate 1% (w/v) (viscosity 98 Pa.s.) in a calcium-free Krebs–Ringer HEPES buffer solution (CF-KRH, pH 7.4). The mixture was poured into a 5 mL plastic syringe and extruded from a 26-gauge stainless steel needle at 3 mL/h. Droplet formation was induced through an electrostatic encapsulation method and a high voltage rate set at 9 kV. The needle was connected to the cathode of a high-voltage DC generator and positioned above the gelation bath, which was connected to a ground wire. The distance between the tip of the needle and the surface of the gelation solution was 20 cm. The droplets were collected in a 100 mM CaCl_2_ solution, where the ionic reaction spontaneously began and crosslinking occurred among Alg molecules to form an ionic gel commonly called an egg-box-like structure. The Alg–Ca MSs were collected via centrifugation after 10 min of soaking in the gelation solution. The mean diameter of the MSs was determined based on the measurement of more than 100 droplets using an optical microscope.

### TGF-β1 encapsulation efficiency

Alg–Ca MSs were dissolved in a sodium citrate (55 mM) solution just after preparation. The encapsulation efficiency of TGF-β1 in Alg–Ca MSs was determined by measuring TGF-β1 via an enzyme-linked immunosorbent assay (ELISA) kit (R&D Systems, Minneapolis, MN) according to the manufacturer’s instructions. Briefly, the solution containing dissolved TGF-β1 was added to pre-coated wells with anti-human TGF-β1 polyclonal antibodies. Then samples were treated with a biotin-conjugated mouse anti-TGF-β1 antibody and streptavidin–horseradish peroxidase, respectively. Color intensity was developed using a tetramethyl benzidine-hydrogen dioxide mixture and terminated with sulfuric acid 0.01 M. The absorbance of each well was determined using a spectrophotometer at 560 nm. We used 50 g MS in 0.5 ml hydrogel. All the procedures were repeated 3 times.

### Release profile of TGF-β1

For this study, three experimental groups were analyzed (Table 1). The final concentration of TGF-β1 in each condition was 10 ng/mL. The prepared specimens were placed in 10 volumes of culture medium (α MEM; Gibco Technologies, Logan, UT) in a low-binding protein tube at 37 °C. The incubating medium was changed at the indicated time intervals. The amount of released TGF-β1 into the media was quantified by an ELISA kit (R&D Systems, Minneapolis, MN). Measurements were performed in triplicate, and the amount of protein release was expressed as a percentage of the initial amount of incorporated TGF-β1. The released TGF-β1 was quantified at the indicated time points over 21 days of incubation.

**Table 1:** Experimental groups utilized in the current study for investigation of release profile of TGF-β1.

No.	Details	Name
1	10 ng *TGF-β1* encapsulated in 1 mL Alg–Ca MS	*TGF-β1*/Alg–Ca MS
2	10 ng *TGF-β1* directly added in 1 mL ECM-derived hydrogel	*TGF-β1*/ECM-derived hydrogel
3	100 ng *TGF-β1* in 1 mL Alg–Ca MSs suspended at a ratio of 1:10 (w/v) in ECM-derived hydrogel	*TGF-β1*/Alg–Ca MS/ECM-derived hydrogel

### Characterization of MSCs

Human bone marrow samples were obtained from 9 patients who were 30–40 years old who underwent a total hip replacement at the Emam Khomeini Hospital following written informed consent. All methods were carried out in accordance with relevant guidelines and regulations. Also, all experimental protocols were approved by Tehran University of Medical Sciences with the licensing committee code number of IR.TUMS.VCR.REC.1396.3152. MSCs were isolated as described in our previous report^23^. Briefly, bone marrow aspirates were diluted with phosphate-buffered saline (PBS), layered over Ficoll solution (Sigma-Aldrich), and centrifuged at 800 g for 20 min to collect mononuclear cells from the gradient interface. Then, mononuclear cells were cultured in Dulbecco’s modified Eagle’s medium–high glucose (DMEM–HG, Gibco, USA) supplemented with 10% fetal bovine serum (FBS, Gibco, USA) and 1% penicillin/streptomycin. MSCs were incubated at 5% CO_2_ and 37 °C until 80% confluence. MSC were used at 3–4th passages for further analyses. They were seeded in tissue culture dishes at 1.0 × 10^3^ cells/cm^2^. Then, the culture medium was changed to osteogenic, chondrogenic, or adipogenic ones for 21 days, as described previously^23^.

## MSCs culture in hydrogel

MSCs viability was investigated by encapsulating them within various decellularized hydrogel groups (Table 2). The ECM-derived hydrogels after neutralization were mixed with MSCs at 1 × 10^6^ cells/mL. 500 µL of hydrogels were poured into a 24-well tissue culture plate and gelled by incubation at 37 °C for 30 min. Then, the cell-laden hydrogel constructs were exposed to culture medium (DMEM–HG) supplemented with 10% fetal calf serum (FCS; Gibco Technologies) and 1% penicillin/streptomycin and incubated at 5% CO_2_ and 37 °C. On days 1, 7, and 21, ECM-derived hydrogels were stained with Calcein AM/propidium iodide (PI) (live/dead).

**Table 2:** Experimental groups utilized in the current study for investigation of MSCs viability.

No.	Details	Name
1	10 ng *TGF-β1* directly added in 1 mL ECM-derived hydrogel	*TGF-β1*/ECM-derived hydrogel
2	neat Alg–Ca MS at ratio of 1: 10 with ECM-derived hydrogel	Alg–Ca MS/ECM-derived hydrogel
3	100 ng *TGF-β1* in 1 mL Alg–Ca MSs suspended at a ratio of 1:10 (w/v) in ECM-derived hydrogel	*TGF-β1*/Alg–Ca MS/ECM-derived hydrogel
4	Neat ECM-derived hydrogel	Negative control

### Ex vivo osteochondral explant and mechanical loading

**Figure 2:**
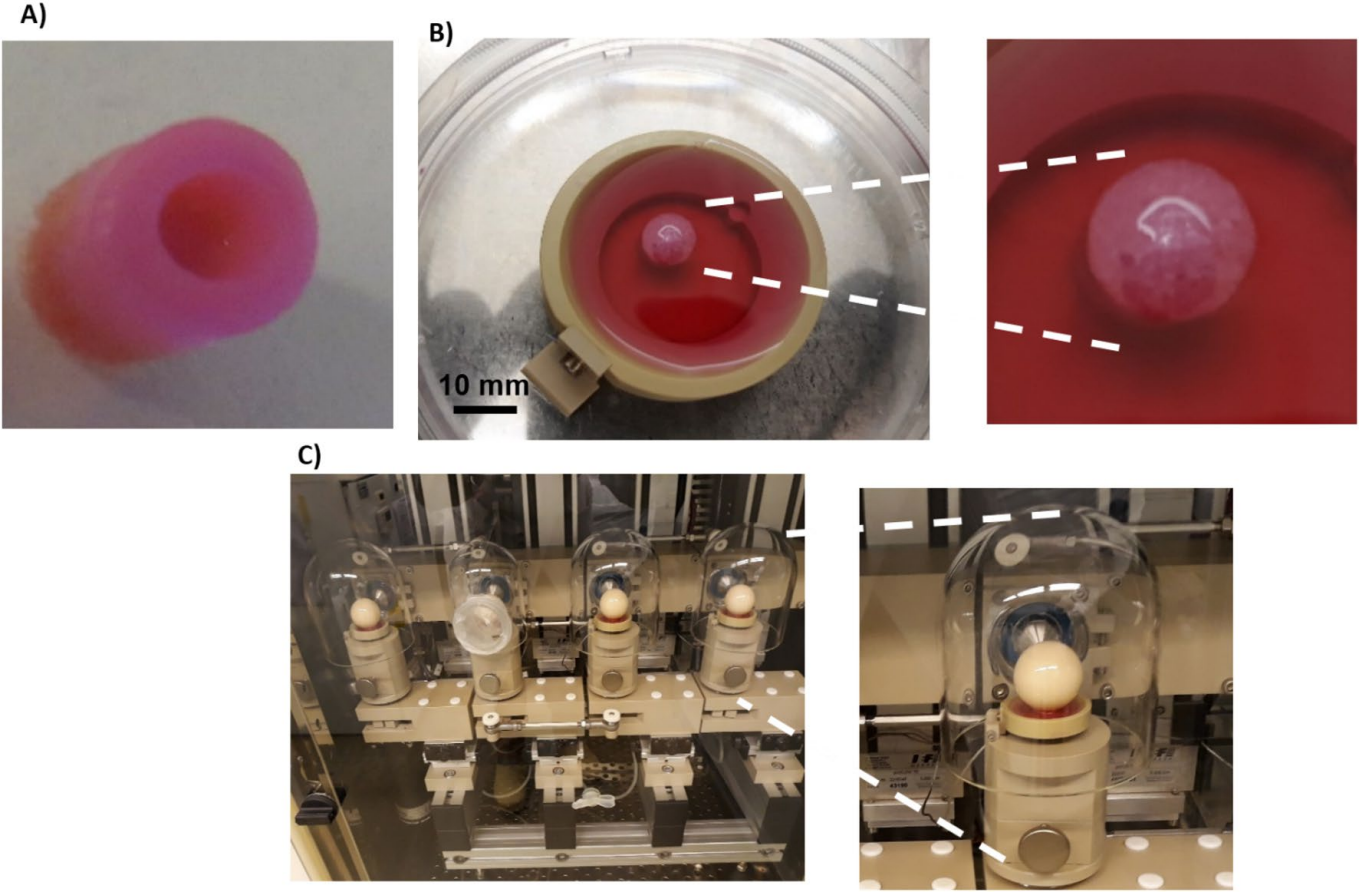
Representative image of osteochondral plug (**A**) before and (**B**) after filling with decellularized matrix derived hydrogel containing MSC placed in PEEK holders for mechanical loading. (**C**) Bioreactor for mechanical conditioning (see method for the detailed loading protocol).

Defects were generated from bovine stifle joint explants using a sterilized 4 mm trephine drill (Brutsch–Ruegger, Urdorf, CH) to centrally remove a full-thickness circular cylindrical cartilage biopsy in dimensions of 4 mm in diameter and 3 mm in depth, as previously described (Figs. 1C, 2A)^24–26^. Afterward, the obtained explants were cultured in DMEM–HG containing 10% FCS, 1% penicillin/streptomycin at 37°C, and 5% CO_2_ for 24 h to ensure the sterility of the explants. Then, specimens were placed in well plates and coated with 1% (w/v) low-gelling agarose (SeaPlaque Agarose, Lonza, Rockland, USA) to prevent cellular outgrowth from the subchondral bone. Osteochondral defects were individually filled with one of the four MSC-laden ECM-derived hydrogels as described above (Fig. 2B).

The specimens were exposed to mechanical stimulation using a custom designed four-station bioreactor, installed in a 5% CO_2_ incubator at 37 °C. The bioreactor concept implies an orthogonally rotating ball, which is pressed onto the explant sample^27^. By oscillating ball rotations, shear motions are generated that reproduce the joint kinematics occurring in vivo. Dynamic compression of the ball onto the sample is applied with linear actuators, while step motors generate the simultaneous oscillation of the ball.

In the current study, the explants were placed into custom sample holders made from PEEK (Fig. 2B). A ceramic hip ball (32 mm in diameter) was pressed onto the osteochondral explants to reach a displacement of 10% of the cartilage height (from the center area) to fully contact the cell-laden hydrogel and the surrounding cartilage (Fig. 2C). After that, loading groups were exposed to axial compression in a sinusoidal manner between 0.4 and 0.8 mm, resulting in an actual strain amplitude of 10–20% of the cartilage explant height at a frequency of 0.5 Hz and simultaneous shear motion by ball oscillation at ± 25° and 0.5 Hz^19^.

The maximal applied mechanical loads corresponded to 15 N, or approximately 0.35 MPa. This type of dynamic axial compression with superimposed sliding motion could more precisely simulate joint articulation compared to axial compression alone^24,28^. The mechanical loading was applied for 1 h per day (23 h without ball contact; free swelling between loading cycles) during 21 consecutive days. The chondrogenic differentiation of loaded MSCs was compared with that of the same cell-laden hydrogel constructs in the absence of mechanical stimulation.

### Chondrogenic induction of MSC-laden hydrogel

The expression of chondrogenic markers in the different ECM hydrogel groups was evaluated by total RNA isolation at day 21 of culture from the ex vivo osteochondral explant model with or without mechanical stimulation. The encapsulated cells were released from the ECM-derived hydrogels by collagenase degradation for 1 h at 37 °C. Then, the total RNA was utilized for the synthesis of the complementary DNA with the first-strand cDNA synthesis kit (PrimeScript™; TaKaRa, Tokyo, Japan). Real-time quantitative PCR was performed on a StepOne™ real-time PCR system (Applied Biosystems, Foster City, CA, USA) using SYBR® Green Supermix (Bio-Rad Laboratories, Hercules, CA, USA). The data were expressed by the 2^−ΔΔCT^ method using StepOne software. Expression of marker genes was normalized to the housekeeping gene glyceraldehyde 3-phosphate dehydrogenase (GAPDH). Primer sequences used for the chondrogenic marker genes, including collagen type II (COLII), collagen type X (COLX), aggrecan (ACAN) and SOX9, are presented in Table 3.

**Table 3:** Oligonucleotide primers used in RT-PCR analysis.

Target gene	Primer Sequence	Accession number	Annealing temperature	Product size
*GAPDH*	F: 5ʹCTCATTTCCTGGTATGACAACGA R: CTTCCTCTTGTGCTCTTGCT	NM_002046.5	60 °C	122 bp
*CoL2a1 (COLII)*	F: ACTCAAGTCCCTCAACAACC R: ATCCAGTAGTCTCCACTCTTCC	NM_001844.4	60 °C	126 bp
*collagen type X alpha 1 chain (COLX)*	F: 5ʹ CATAAAAGGCCCACTACCCAAC 3ʹ R: 5ʹ ACCTTGCTCTCCTCTTACTGC 3ʹ	NM_000493.3	60 °C	91 bp
*Aggrecan (ACAN)*	F: 5ʹCTGGACAAGTGCTATGCCG 3ʹ R: 5ʹGAAGGAACCGCTGAAATGC 3ʹ	NM_001135.3	60 °C	191 bp
*SOX9*	F:5ʹCCCTTCAACCTCCCACACTAC3ʹ R:5ʹGCTGTGTGTAGACGGGTTGTT3ʹ	NM_000346.3	60 °C	253 bp

### Histological analysis

The osteochondral defect (including the different ECM hydrogels) or the ECM hydrogels cultured alone were fixed in 70% methanol for 24 h, and then they were incubated for decalcification in EDTA 10% for 15 days, followed by paraffin embedding. Histological section (7 μm thickness) were stained with toluidine blue to assess nuclei and hydrogel deposition in the defect region.

#### SEM preparation

Cell-laden and cell-free ECM-derived hydrogels were prepared for SEM analysis after 7 days of incubation in 4% glutaraldehyde. Then, the samples were dehydrated with ascending grades of ethanol: 30, 40, 60, 70, 80, 90, and 95%. Afterward, the samples were coated with a gold layer. Microphotographs were obtained by scanning electron microscopy using a VEGA, TESCAN apparatus (Czech Republic).

#### Biochemical analysis: s-GAG and DNA content

Cell-hydrogel constructs removed from osteochondral defect models in the loaded and unloaded groups were collected for biochemical analysis. Cell-hydrogel constructs were digested overnight in 0.5 mg/mL proteinase K at 56 °C (2.5 U/mg, chromozyme assay; Roche, Mannheim, Germany). DNA content was measured using the QUANT-iT Picogreen, DS assay kit (Molecular Probes, Life Technologies). The total amounts of sulfated glycosaminoglycan (s-GAG) were determined by the dimethyl-methylene blue (DMMB) dye-binding assay^25,26^.

### Statistical analysis

Statistical analysis was performed using SPSS 17.0 software (SPSS, USA). Three different samples within the same experiment were examined for checking the reproducibility. All data are expressed as mean ± SD. A one-way ANOVA with a Tukey post hoc test was used to determine differences among the four groups, and an independent *T*-test was used to determine differences among two groups (*p* < 0.05 was considered statistically significant).

## Results

### Hydrogel microstructure and cell morphology

**Figure 3:**
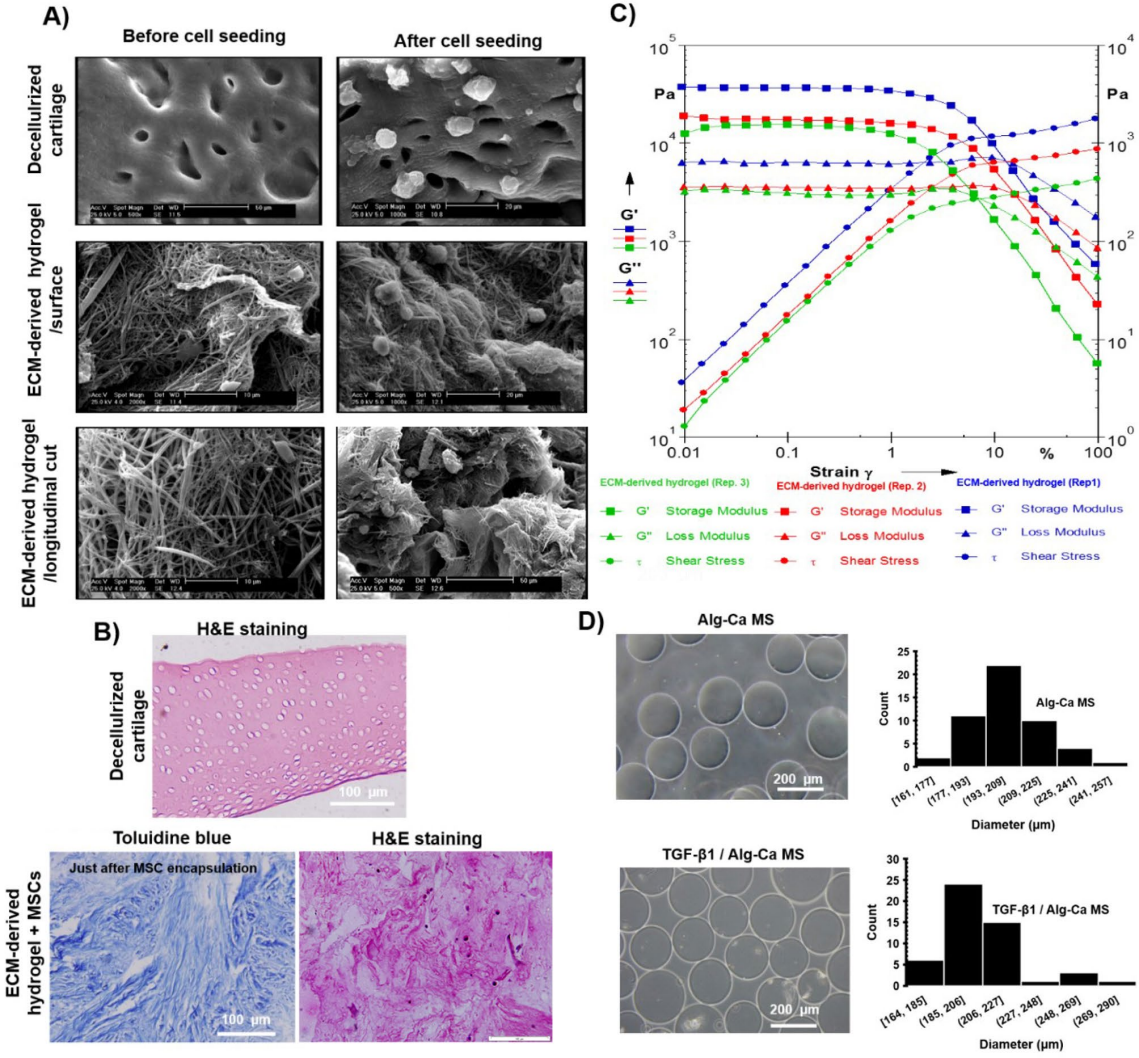
(**A**) SEM images of the decellularized cartilage and decellularized hydrogels before and after cell seeding on days 0 and 7, respectively (**B**) H&E and toluidine blue staining of decellularized cartilage and MSCs encapsulated in ECM-derived hydrogel (day 0) (**C**) Rheology test of hydrogels was done at replicates (Rep.) of 1, 2, and 3 (n = 3) (**D**) Microphotographs and size distribution of Alg–Ca and TGF-β1/Alg–Ca MS droplets.

The SEM images of the decellularized cartilage or ECM-derived hydrogel surfaces, as well as longitudinal cross-sections before and after cell encapsulating, are shown in Fig. 3A. The SEM photographs revealed preservation of the native structures of ECM and a uniform fibrillar structure of the ECM-derived hydrogel. Moreover, a porous three-dimensional microstructure with interconnected pores composed of oriented and dense intermeshed fibers is shown, indicating a potentially favorable microenvironment for cell attachment and growth ^9^. The encapsulated cells in decellularized cartilage and ECM-derived hydrogels attached and remained round in shape or with an elliptic morphology (Fig. 3A), which implies an inherent cell-friendly property of the hydrogels^9^.

Besides, H&E staining also showed that cartilage tissues were successfully decellularized (Fig. 3B), and DNA content was obtained at 0.03 ± 0.005 ng/mg of cartilage tissue of dry weight, which indicates the necessity of chemical treatment with SDS at lower concentrations as well as physical treatment using Snap freezing and mechanical force for proper decellularization of tissue. Also, encapsulated MSCs within the ECM-derived hydrogel stained with toluidine blue at day 0 are shown in Fig. 3B. The storage modulus (Gʹ) and loss modulus (Gʹʹ) of the cylindrical hydrogel did not change until 1% strain, and ECM-derived hydrogels showed linear viscoelasticity properties (Fig. 3C). In general, the storage modulus was higher than the loss modulus, indicating the dominant elastic characteristics of these hydrogels. The maximum storage modulus reached 30 kPa at 37 °C for ECM-derived hydrogel (Fig. 3C).

### Characterization of TGF-β1/Alg–Ca MS

We evaluated the size distribution and diameter of Alg–Ca MSs to assess the reproducibility and uniformity of MSs for TGF- β1 encapsulation. As expected, Alg–Ca MSs could be obtained using the electrostatic system (Fig. 3D), as previously reported^29,30^. A narrow size range and size consistency among the created MSs are shown by the quantification of MS diameter, which revealed average sizes of 206 ± 17 µm and 202 ± 8 µm for Alg–Ca and TGF-β1/Alg–Ca MSs, respectively (Fig. 3D). These results agreed with previous studies and proved that incorporation of TGF-β1 in the Alg–Ca solution did not hinder particle sphericity and size distribution due to the opposing charges between the molecules as well as ionic gelation^29,30^. The narrow size distribution of the MSs was confirmed by the coefficients of variance (defined as a standard deviation divided by the average diameter of microspheres) of less than 5.4% and 7.9% for Alg–Ca MS and TGF-β1/Alg–Ca MS, respectively. The diameter of Alg–Ca MS was relatively uniform, as indicated by the coefficient of variance for the size distribution under 8%.

### Release of encapsulated TGF-β1

**Figure 4:**
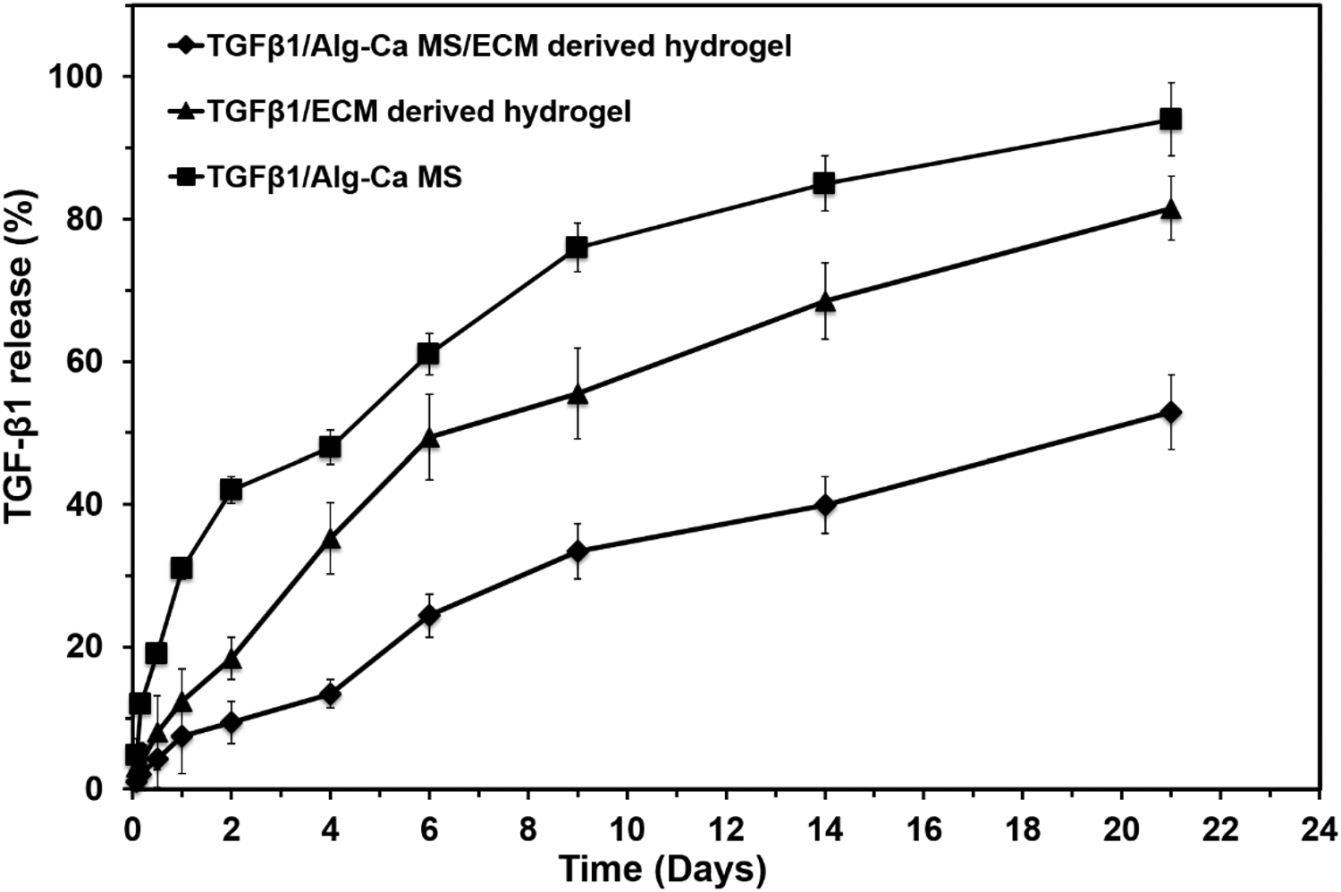
Characterization of the in vitro release profile of *TGF-β1* loaded Alg–Ca MS and ECM-derived hydrogel. Cumulative release is calculated from the total amount of *TGF-β1* released over 21 days. Four replicates were considered in each test.

Modulation of bioactive molecule delivery is recognized as a crucial factor for the applicability of scaffolds in tissue engineering and regenerative medicine^21,22^. The bioactive release profiles of TGF-β1 from the TGF-β1/Alg–Ca MS and TGF-β1/Alg–Ca MS/ECM-derived hydrogels are presented in Fig. 4. The amount of TGF-β1 in the supernatant of the solution was then measured, and encapsulation efficacy was determined at 79% ± 4.1%. The high degree of efficacy could be due to the availability of positive charge amino groups in TGF-β1 which could interact via -OH and carbonyl groups of Alg, forming an amide hydrogen bond, and in following TGF-β1/Alg–Ca availability, proceeded with ionic interaction with CaCl_2_.

An increasing release rate of TGF-β1 of more than 31% was observed within the initial 12 h of encapsulation in TGF-β1/Alg–Ca MS (Fig. 4). Fast release continued at 42% at 2 days, and cumulative release of TGF-β1 continued up to 94% at 21 days. This could be the consequence of the dissociation of Alg–Ca bonds due to the substitution by sodium or potassium ions present in culture media^30,31^.

The TGF-β1 release from TGF-β1/Alg–Ca MS/ECM-derived hydrogel was initially very low, 7% at day 2, and then reached about 32% at day 9, which represents a lower release compared to 51% of TGF-β1/Alg–Ca MS and 77% of TGF-β1/ECM-derived hydrogel (Fig. 4). The release rate from TGF-β1/Alg–Ca MS/ECM-derived hydrogel continued steadily until 21 days, at 52%. The TGF-β1/ECM-derived hydrogel groups showed an intermediate release rate (Fig. 4**)**. This result indicated that encapsulation of TGF-β1 in Alg–Ca MS/ECM or in ECM alone hydrogels could provide reliable systems for sustainable delivery, and using both structures resulted in the most controllable release approach for sustained release of TGF-β1 as a bioactive substrate required for cell and cartilage tissue development.

### Cellular viability in 3D hydrogels

**Figure 5:**
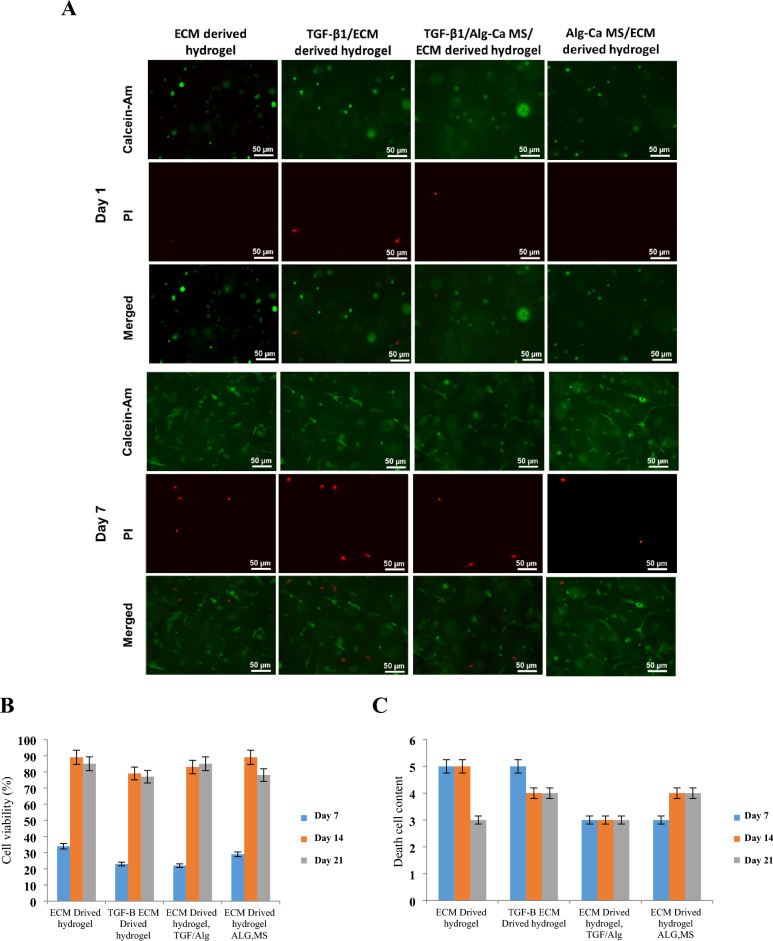
(**A**) Images showing cell viability of MSC in the different ECM-derived hydrogels stained with Calcein Am/propidium iodide (PI) (live/dead). Hydrogels were encapsulated until 21 days. Only a few dead cells could be observed within the hydrogel up to 7 days, while the diagrams reveal more details on various groups. (**B**) Live cell content in various groups on 7, 14, and 21 days (**C**) Death cell content in various groups on 7, 14, and 21 days.

The viability of the MSCs within the ECM-derived hydrogels containing TGF-β1 and Alg–Ca MS was evaluated using the fluorescence microscope. The samples were stained with Calcein AM/ propidium iodide (PI) after up to 21 days of culture. Cells at day 1 after encapsulation were viable and spherical (Fig. 5). Cell viability was excellent up to 21 days of culture, and MSCs could elongate and spread (white arrows). There were no significant differences between the various hydrogels.

### Dimensions of osteochondral explants

**Figure 6:**
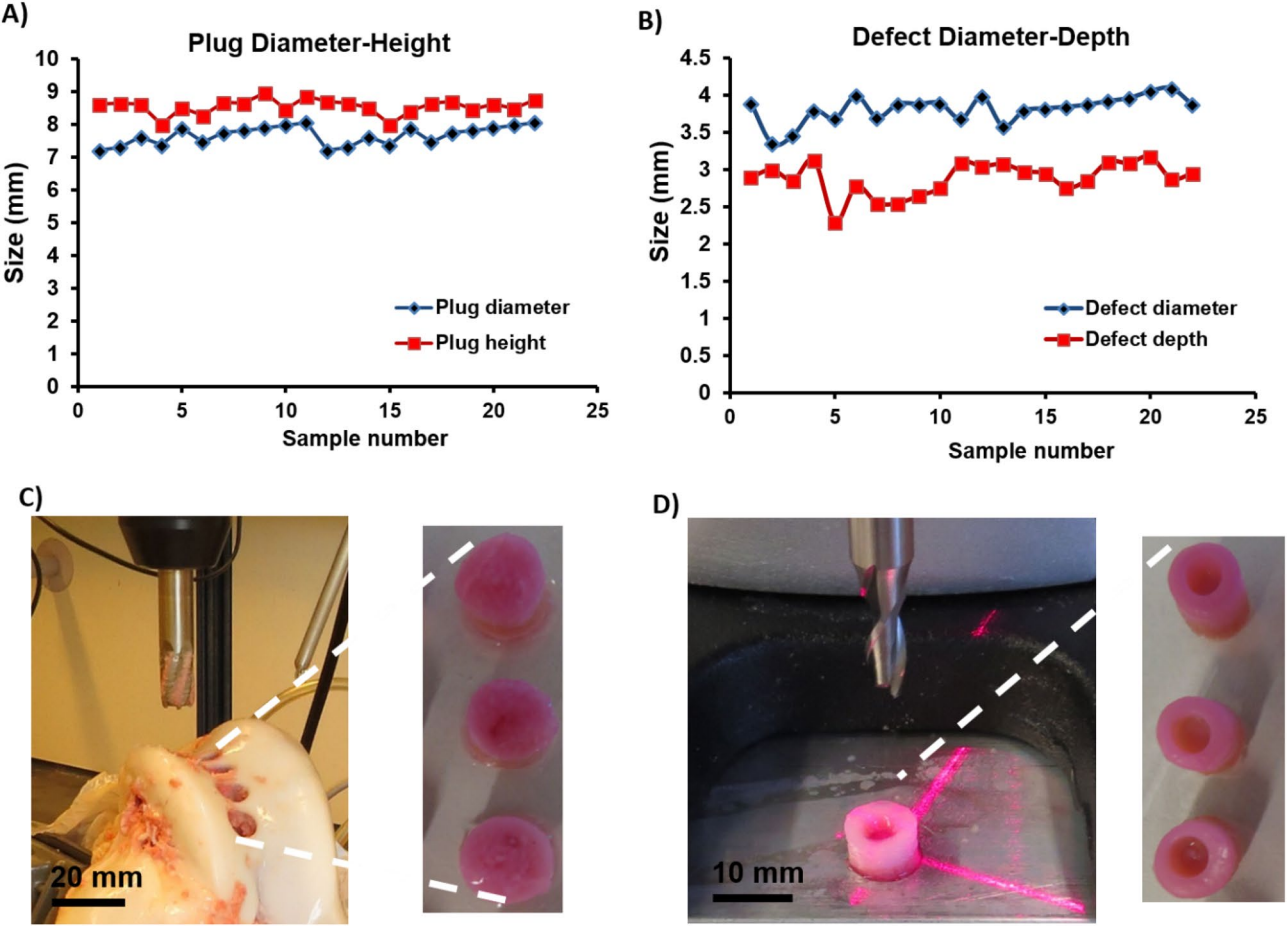
Size distribution of (**A**) osteochondral plug and (**B**) defect, which indicate good reproducibility of osteochondral plugs. (**C**) Representative image of an osteochondral explant production using the compact drill press. (**D**) The trephine is adjusted to create the desired depth of the circular groove, which was adjusted by the digital drilling press.

The generation of the chondral defect model was validated by repeated measurements of the osteochondral explants, and the reproducibility of the produced chondral defect was similar to the previous reported parameters ^26^. Briefly, explants from calf stifle joints showed an average diameter and height of 7.65 ± 15 mm and 9.34 ± 12 mm, respectively (Fig. 6A,C). The diameter and depth of the chondral defects generated in the middle of the explants showed a diameter of 3.82 ± 0.13 mm and a depth of 2.72 ± 0.12 mm (Fig. 6B,D).

### Chondrogenic gene expression for ex vivo osteochondral explants

**Figure 7:**
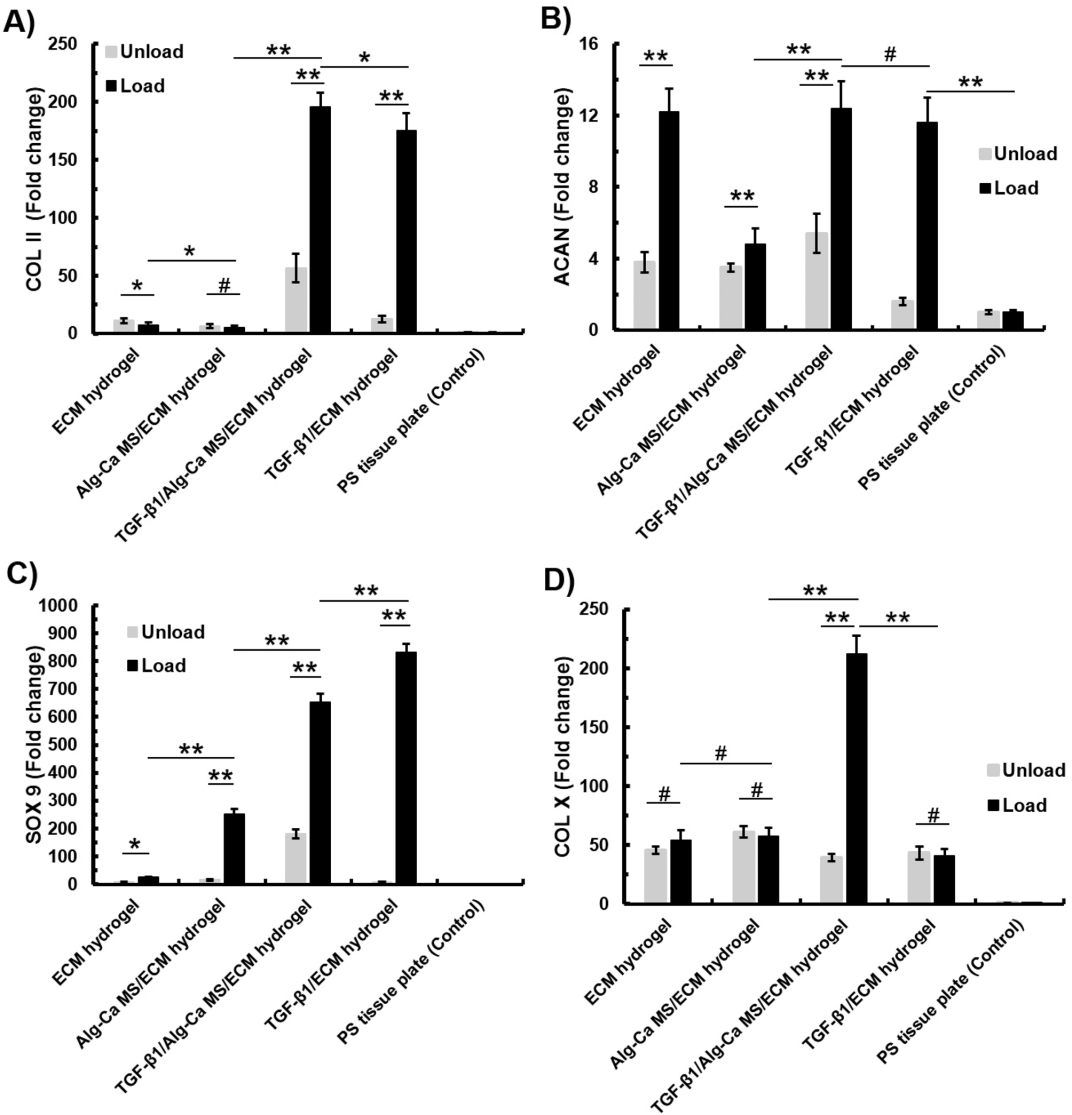
Fold change of (**A**) *collagen type II (COL ӀӀ)*, (**B**) *aggrecan (ACAN)*, (**C**) *SOX9* and (**D**) *Collagen type X (COL X)* expression for encapsulated MSCs in hydrogels at day 21, compared with encapsulated cells in ECM hydrogel just after encapsulation. Hydrogels were implanted in created defect plug as an ex vivo model and underwent mechanical load until 21 days. Polystyrene (PS) tissue plate used as control. (Mean ± SD; n = 4; *: *p* < 0.05, **: *p* < 0.01, #: *p* > 0.05).

Results for COLII, ACAN, SOX9, and COLX gene expression are shown in Fig. 7. The results are normalized to MSCs cultured in 2D at day 0. In non-loaded TGF-β1/ECM-derived hydrogel conditions, the level of COLII expression did not change in compared to ECM hydrogel and Al–Ca Ms/ECM-derived hydrogel groups. Meanwhile, the TGF-β1/Alg–Ca MS/ECM-derived hydrogel condition showed increased COLII expression compared to the other groups. Interestingly, in gels containing TGF-β1/ECM-derived hydrogel under mechanical loading experimental conditions, COLII expression was increased (Fig. 7A).

Regarding ACAN expression level, in non-loaded conditions, the presence of TGF-β1 did not show any significant changes among groups. On the contrary, with mechanical loading, ACAN expression levels were significantly increased in all hydrogels, although this boost was limited in the Al–Ca MS/ECM hydrogel group (Fig. 7B). For SOX9, the highest expression level was observed for TGF-β1/ECM-derived hydrogel under mechanical load (Fig. 7C). The COLX did not show changes in gene expression levels in all conditions, except for a clear increase in the TGF-β1/Alg–Ca MS/ECM-derived hydrogel group under mechanical load (Fig. 7D).

### Cellular growth and GAG content in ex vivo experiments

**Figure 8:**
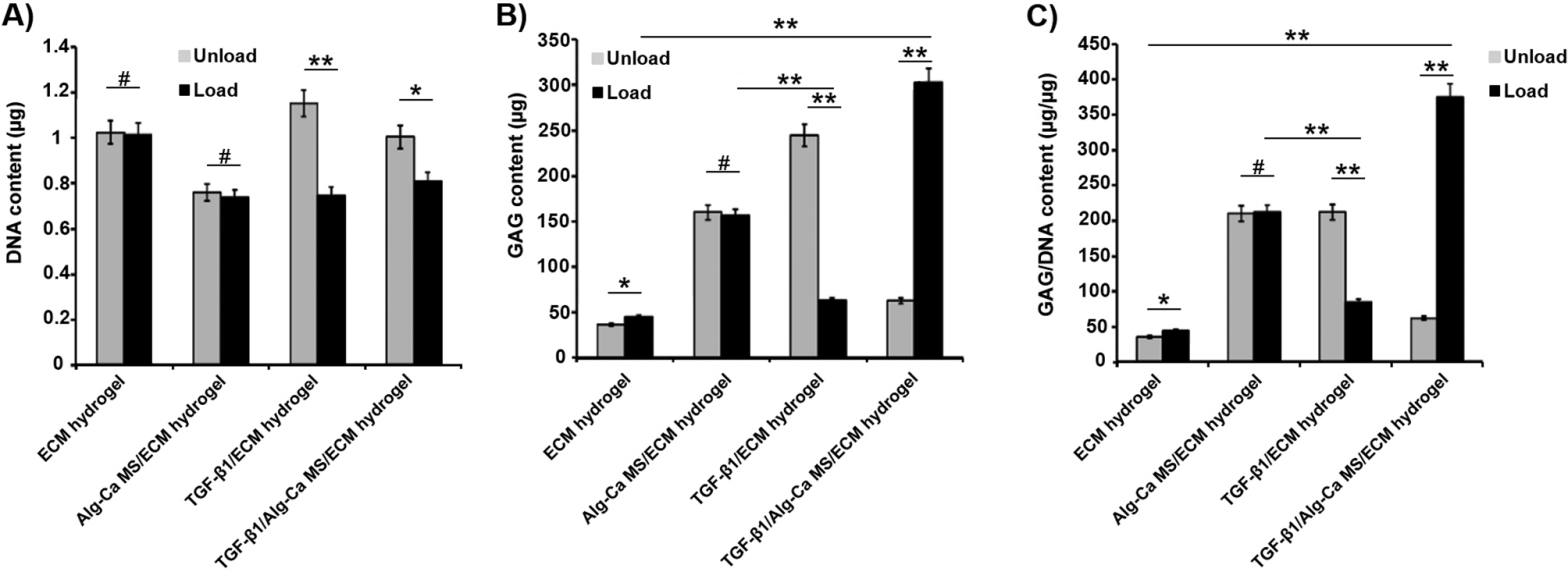
(**A**) DNA content, (**B**) GAG content, and (**C**) the ratio of GAG/DNA at day 21. (Mean ± SD; n = 4; *: *p* < 0.05, **: *p* < 0.01 and #: *p* > 0.05).

The DNA content showed significant differences among loaded and unloaded groups in the TGF-β1 containing groups, with higher DNA amounts in the unloaded samples (Fig. 8A). There was a noticeable drop in the GAG content in the TGF-β1/ECM hydrogel group when mechanical load was applied (Fig. 8B). Interestingly, the maximum levels of GAG and GAG/DNA content were observed in TGF-β1/Alg–Ca MS/ECM-derived hydrogel under loading conditions (Fig. 8B,C).

### Histological observations of the ex vivo condition

**Figure 9:**
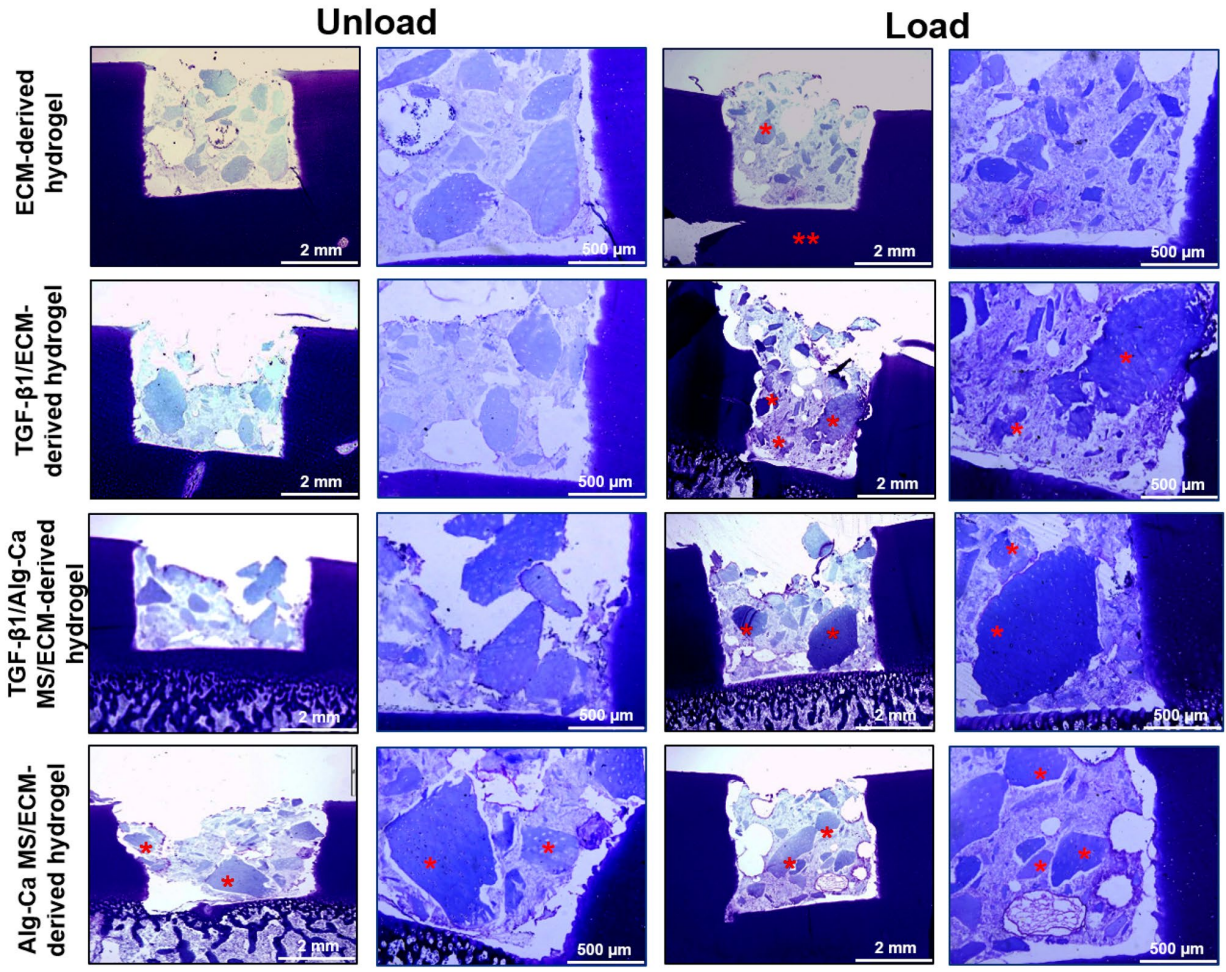
Toluidine blue staining of MSCs encapsulated in ECM-derived hydrogels and implanted in chondral defect for 21 days. Horizontal scale bars are 2 mm and in high magnification 500 µm. Asterisks show the cartilage like pieces which seems new cartilage formation.

Histological observations of the ex vivo condition depicted that the integration of the developed implants and the defect area is acceptable (Fig. 9). Furthermore, the color intensity and volume of the staining appeared greater for hydrogels containing TGF-β1 in both loading and non-loading conditions (Fig. 9). Nevertheless, these observations could only be considered very preliminary due to the lack of any quantification process. Results showed that the loaded specimens demonstrated a higher cartilage formation compared to the unload specimens.

## Discussions

Previously, researchers are believed that healing cartilage defects such as osteoarthritis and rheumatoid arthritis were challenging and difficult to achieve^32,33^. Also, previous studies showed that mechanical stimuli are vital for developing and maintaining articular cartilage^25,28^. Researchers demonstrated that various mechanical stimuli are associated with the cartilage microenvironment, including hydrostatic, tension, compression, and shear^26,28^. Bioreactors can mimic physiological loading conditions of the natural tissue microenvironment and have been used to investigate cartilaginous tissue formation in vitro^25,28^.

Recalling that hydrogels are proper a class of materials that could be used in various tissue engineering applications^34,35^; here, a decellularized cartilage hydrogel containing Alg–Ca MS loaded with TGF-β1 for prolonged control release of this factor was investigated for its chondrogenic differentiation potential of MSC in in vitro and ex vivo models. The utilized bioreactor provided mechanical stress utilizing a moving and rolling ceramic ball (Fig. 1). This type of bioreactor would mimic compressive and shear forces approximating the kinematic motion of an articulating joint to determine whether applied forces affected stem cells’ fate^36^.

Despite similar amounts of DNA among the different experimental groups, we observed a significant increase in GAG production for TGF-β1/Alg–Ca MS/ECM-derived hydrogel after multi-axial mechanical load compared to the other groups. In addition, the TGF-β1 encapsulated in the Alg–Ca MSs provided sustained release of TGF-β1 up to 21 days which showed control release of TGF-β1 was effective on the quality of cartilage preservation. This is indicating that Alg–Ca MS retention of TGF-β1 is beneficial to obtain a longer and beneficial period of this growth factor for cartilage tissue engineering. This is in line with previous work where continuous mechanical load and presence of TGF-β1 induced a significant increase in the expression of chondrogenic markers and GAG content^32,37,38^.

However, the presence of TGF-β1 is also associated with an increase of the hypertrophic marker, type X collagen. In this context, we speculated that this hypertrophic driving effect of TGF-β1, could be due to its prolonged released and its long-term stimulation on the surrounding cells in the TGF-β1/Alg–Ca MS/ECM-derived hydrogel. As shown in Fig. 7, all the other hydrogel compositions did not induce type X collagen, even in presence of TGF-β1. From this point of view, the TGF-β1/Alg–Ca MS/ECM hydrogel looks the most promising, with respect to increase the chondrogenic markers and GAG/DNA ratio, while keeping a limited induction of hypertrophy. Although, it is presently difficult to suggest a proper reason to this observation, except for the presence of an increase in calcium ions.

It should be noted that as this work was a study on the differentiation of MSCs to chondrocytes, investigating the hypertrophy in the differentiated cells was not the aim of this study, while some proper markers among four previously mentioned genes; COL II, ACAN, SOX 9, and COL X could be used for this analysis. Studies showed that a promoted cell differentiation to chondrocytes could be occurred, characterized by increased expressions of COL X, MMP-13 and Runx2^39^. Meanwhile, researchers noted that SOX 9, aggrecan and COL II expressions were down-regulated^39^. Accordingly, COL X is associated with MMP-13, and are expressed simultaneously^40^.

Although mechanical loading on the TGF-β1/ECM hydrogel has increased the expression of COL II, ACAN, and SOX9 significantly, the DNA and GAG contents of this hydrogel has been decreased after mechanical loading. Recalling that GAG results are representative of protein expression, while Col II, ACAN, and SOX9 show gene expression, differences among them is justified. Moreover, as their expression were measured via two separated experiments, it is noticeable that the experiment conditions may be altered. The other effective factor in this reverse trend is the un-encapsulating the TGF-β1 in the hydrogel. Studies showed that prompt releasing of growth factors could alter the proteins expression, effecting their signaling pathways^41^. However, our targeted group, TGF-β1/Alg–Ca MS/ECM-derived hydrogel, shows the same trend in gene and protein expression, demonstrating the acceptable efficiency of encapsulating the TGF-β1 in Alg–Ca MSs, and sustain releasing of them into ECM-derived hydrogel, promoting the proliferation and differentiation of MSCs appropriately.

The viability staining showed the presence of alive encapsulated MSCs in a uniform and homogenous distribution within the different hydrogels. Because of the nature of our hydrogels, which contain decellularized ECM and some of them alginate, it was difficult to quantify the Toluidine blue staining observed in Fig. 9. Other quantification methods, like S^35^ labeling of the newly synthesized proteoglycans, will need to be applied in future works.

## Conclusions

In this work, we investigated the controlled release of TGF-β1 as an active mediator of MSCs in a biocompatible scaffold and mechanical stimulation for cartilage tissue engineering. For this aim, ECM-derived hydrogel containing alginate-based MSs loaded with TGF-β1 was developed for the improvement of chondrogenic differentiation of human MSCs. Moreover, physiological conditions were simulated by using ex vivo explants and a complex multiaxial loading bioreactor. Our results recommended that in the presence of applied mechanical stimuli and prolonged delivery of TGF-β1, the chondrogenic genes were upregulated compared to unloaded osteochondral constructs, which could exert substantial effects in biomimetic cartilage tissue formation. Prolonged TGF-β1 retention using Alg–Ca MS remains beneficial to obtain a more extended period growth factor in cartilage tissue engineering, while mechanical loading which mimics native existing pressure and force on cartilage had most effective impact on the expression of chondrogenic factors. Therefore, it is expected that the simultaneous implication of controlled delivery of growth factors and periodic mechanical force could synergistically induce cartilage tissue engineering and regeneration.

## Data Availability

All data generated or analyzed during this study are included in this published article.

## Abbreviations

ACAN: Aggrecan
ACI: Autologous chondrocyte implantation
COLII: Collagen type II
COLX: Collagen type X
DMMB: Dimethyl-methylene blue
DMEM–HG: Dulbecco’s modified Eagle’s medium–high glucose
ECM: Extracellular matrix
FBS: Fetal bovine serum
FCS: Fetal calf serum
GAPDH: Glyceraldehyde 3-phosphate dehydrogenase
MSCs: Mesenchymal stromal cells
GAPDH: Glyceraldehyde 3-phosphate dehydrogenase
MSs: Microspheres
PBS: Phosphate-buffered saline
PI: Propidium iodide
SEM: Scanning electron microscopy
s-GAG: Sulfated glycosaminoglycan
TGF-β1: Transforming growth factor beta

## References

[1] Fusco M, Skaper SD, Coaccioli S, Varrassi G, Paladini A (2017). Degenerative joint diseases and neuroinflammation. Pain Pract..

[2] Yousefi AM, Hoque ME, Prasad RG, Uth N (2015). Current strategies in multiphasic scaffold design for osteochondral tissue engineering: a review. J. Biomed. Mater. Res. Part A.

[3] Azami M, Beheshtizadeh N (2021). Identification of regeneration-involved growth factors in cartilage engineering procedure promotes its reconstruction. Regen. Med..

[4] Xu, Y. *et al.* Unraveling of advances in 3D-printed polymer-based bone scaffolds. *Polymers* **14**(3), 566. 10.3390/polym14030566 (2022). 10.3390/polym14030566 PMC8840342 35160556

[5] Solanki K, Shanmugasundaram S, Shetty N, Kim S-J (2021). Articular cartilage repair and joint preservation: A review of the current status of biological approach. J. Clin. Orthop. Trauma.

[6] Vaish A, Shanmugasundaram S, Kim SA, Lee D-H, Shetty AA, Kim SJ (2022). Biological reconstruction of the joint: Concepts of articular cartilage regeneration and their scientific basis. J. Clin. Orthop. Trauma.

[7] Kim YS, Majid M, Melchiorri AJ, Mikos AG (2019). Applications of decellularized extracellular matrix in bone and cartilage tissue engineering. Bioeng. Transl. Med..

[8] Yuan J, Wang Y, Yang W, Li X, Tao K, He W, Yan J (2023). Biomimetic peptide dynamic hydrogel inspired by humanized defensin nanonets as the wound-healing gel coating. Chem. Eng. J..

[9] Bordbar S, Lotfi Bakhshaiesh N, Khanmohammadi M, Sayahpour FA, Alini M, Baghaban Eslaminejad M (2020). Production and evaluation of decellularized extracellular matrix hydrogel for cartilage regeneration derived from knee cartilage. J. Biomed. Mater. Res. Part A..

[10] Szychlinska MA, Stoddart MJ, D’Amora U, Ambrosio L, Alini M, Musumeci G (2017). Mesenchymal stem cell-based cartilage regeneration approach and cell senescence: Can we manipulate cell aging and function?. Tissue Eng. Part B Rev..

[11] Cao J, Chen C, Wang Y, Chen X, Chen Z, Luo X (2016). Influence of autologous dendritic cells on cytokine-induced killer cell proliferation, cell phenotype and antitumor activity in vitro. Oncol. Lett..

[12] Tekari A, Luginbuehl R, Hofstetter W, Egli RJ (2015). Transforming growth factor beta signaling is essential for the autonomous formation of cartilage-like tissue by expanded chondrocytes. PLoS ONE.

[13] Yu Y, Wang L, Ni S, Li D, Liu J, Chu HY, Zhang N, Sun M, Li N, Ren Q, Zhuo Z, Zhong C, Xie D, Li Y, Zhang ZK, Zhang H, Li M, Zhang Z, Chen L, Pan X, Xia W, Zhang S, Lu A, Zhang BT, Zhang G (2022). Targeting loop3 of sclerostin preserves its cardiovascular protective action and promotes bone formation. Nat. Commun..

[14] Huey DJ, Hu JC, Athanasiou KA (2012). Unlike bone, cartilage regeneration remains elusive. Science.

[15] Luo G, Zhou Z, Huang C, Zhang P, Sun N, Chen W, Deng C, Li X, Wu P, Tang J, Qing L (2023). Itaconic acid induces angiogenesis and suppresses apoptosis via Nrf2/autophagy to prolong the survival of multi-territory perforator flaps. Heliyon.

[16] Wang Q, Tan QY, Xu W, Qi HB, Chen D, Zhou S, Ni ZH, Kuang L, Guo JY, Huang JL, Wang XX, Wang ZQ, Su N, Chen L, Chen B, Jiang WL, Gao Y, Chen HG, Du XL, Xie YL, Chen L (2017). Cartilage-specific deletion of Alk5 gene results in a progressive osteoarthritis-like phenotype in mice. Osteoarthr. Cartil..

[17] van der Kraan PM (2018). Differential role of transforming growth factor-beta in an osteoarthritic or a healthy joint. J. Bone Metab..

[18] Venkatesan JK, Frisch J, Rey-Rico A, Schmitt G, Madry H, Cucchiarini M (2017). Impact of mechanical stimulation on the chondrogenic processes in human bone marrow aspirates modified to overexpress sox9 via rAAV vectors. J. Exp. Orthop..

[19] Li X, Ding W, Wang S, Yang L, Yu Q, Xiao C, Chen G, Zhang L, Guan S, Sun D (2023). Three-dimensional sulfated bacterial cellulose/gelatin composite scaffolds for culturing hepatocytes. Cyborg Bionic Syst..

[20] Ciofani G, Raffa V, Menciassi A, Micera S, Dario P (2007). A drug delivery system based on alginate microspheres: Mass-transport test and in vitro validation. Biomed. Microdevices.

[21] DeFail AJ, Chu CR, Izzo N, Marra KG (2006). Controlled release of bioactive TGF-β1 from microspheres embedded within biodegradable hydrogels. Biomaterials.

[22] Kim J, Lin B, Kim S, Choi B, Evseenko D, Lee M (2015). TGF-β1 conjugated chitosan collagen hydrogels induce chondrogenic differentiation of human synovium-derived stem cells. J. Biol. Eng..

[23] Safari F, Fani N, Eglin D, Alini M, Stoddart MJ, Baghaban Eslaminejad M (2019). Human umbilical cord-derived scaffolds for cartilage tissue engineering. J. Biomed. Mater. Res. A..

[24] Grad S, Loparic M, Peter R, Stolz M, Aebi U, Alini M (2012). Sliding motion modulates stiffness and friction coefficient at the surface of tissue engineered cartilage. Osteoarthr. Cartil..

[25] Jeon JE, Schrobback K, Hutmacher DW, Klein TJ (2012). Dynamic compression improves biosynthesis of human zonal chondrocytes from osteoarthritis patients. Osteoarthr. Cartil..

[26] van Schaik TJ, Gaul F, Dorthé EW, Lee EE, Grogan SP, D’Lima DD (2018). Development of an ex vivo murine osteochondral repair model. Cartilage.

[27] Wimmer MA, Grad S, Kaup T, Hänni M, Schneider E, Gogolewski S, Alini M (2004). Tribology approach to the engineering and study of articular cartilage. Tissue Eng..

[28] Vainieri M, Wahl D, Alini M, van Osch G, Grad S (2018). Mechanically stimulated osteochondral organ culture for evaluation of biomaterials in cartilage repair studies. Acta Biomater..

[29] Khanmohammadi M, Sakai S, Taya M (2019). Characterization of encapsulated cells within hyaluronic acid and alginate microcapsules produced via horseradish peroxidase-catalyzed crosslinking. J. Biomater. Sci. Polym. Ed..

[30] Khanmohammadi M, Zolfagharzadeh V, Bagher Z, Soltani H, Ai J (2020). Cell encapsulation in core-shell microcapsules through coaxial electrospinning system and horseradish peroxidase-catalyzed crosslinking. Biomed. Phys. Eng. Express.

[31] Bahrami N, Bayat M, Farzin A, Sadredin Hajseyedjavadi M, Goodarzi A, Salehi M, Karimi R, Mohamadnia A, Ahmadi A, Khanmohammadi M (2019). The ability of 3D alginate/polyvinyl alcohol cross-linked hybrid hydrogel to differentiate periodontal ligament stem cells into osteoblasts. Arch. Neurosci..

[32] Schätti O, Grad S, Goldhahn J, Salzmann G, Li Z, Alini M, Stoddart M (2011). A combination of shear and dynamic compression leads to mechanically induced chondrogenesis of human mesenchymal stem cells. Eur. Cell Mater..

[33] Chen S, Li Y, Zhi S, Ding Z, Huang Y, Wang W, Zheng R, Yu H, Wang J, Hu M, Miao J, Li J (2020). lncRNA Xist regulates osteoblast differentiation by sponging miR-19a-3p in aging-induced osteoporosis. Aging Dis..

[34] Shen W, Pei P, Zhang C, Li J, Han X, Liu T, Shi X, Su Z, Han G, Hu L, Yang K (2023). A polymeric hydrogel to eliminate programmed death-ligand 1 for enhanced tumor radio-immunotherapy. ACS Nano.

[35] Khan MUA, Stojanović GM, Abdullah MFB, Dolatshahi-Pirouz A, Marei HE, Ashammakhi N, Hasan A (2024). Fundamental properties of smart hydrogels for tissue engineering applications: A review. Int. J. Biol. Macromol..

[36] Li J, Hua X, Jones AC, Williams S, Jin Z, Fisher J, Wilcox RK (2016). The influence of the representation of collagen fibre organisation on the cartilage contact mechanics of the hip joint. J. Biomech..

[37] Li Z, Kupcsik L, Yao SJ, Alini M, Stoddart MJ (2010). Mechanical load modulates chondrogenesis of human mesenchymal stem cells through the TGF-β pathway. J. Cell. Mol. Med..

[38] Terraciano V, Hwang N, Moroni L, Park HB, Zhang Z, Mizrahi J, Seliktar D, Elisseeff J (2007). Differential response of adult and embryonic mesenchymal progenitor cells to mechanical compression in hydrogels. Stem Cells.

[39] Xiao D, Bi R, Liu X, Mei J, Jiang N, Zhu S (2019). Notch signaling regulates MMP-13 expression via Runx2 in chondrocytes. Sci. Rep..

[40] Borzí RM, Olivotto E, Pagani S, Vitellozzi R, Neri S, Battistelli M, Falcieri E, Facchini A, Flamigni F, Penzo M, Platano D, Santi S, Facchini A, Marcu KB (2010). Matrix metalloproteinase 13 loss associated with impaired extracellular matrix remodeling disrupts chondrocyte differentiation by concerted effects on multiple regulatory factors. Arthritis Rheum..

[41] Itatani Y, Kawada K, Sakai Y (2019). Transforming growth factor-β signaling pathway in colorectal cancer and its tumor microenvironment. Int. J. Mol. Sci..

